# Comparative Anatomy of the Trabecular Meshwork, the Optic Nerve Head and the Inner Retina in Rodent and Primate Models Used for Glaucoma Research

**DOI:** 10.3390/vision1010004

**Published:** 2016-07-18

**Authors:** Liwen Chen, Yin Zhao, Hong Zhang

**Affiliations:** Department of Ophthalmology, Tongji Hospital, Tongji Medical College, Huazhong University of Science and Technology, 1095 Jiefang Avenue, 430030 Wuhan, China

**Keywords:** morphology, trabecular meshwork, lamina cribrosa, optic nerve head, animal models

## Abstract

Glaucoma is a heterogeneous group of ocular disorders with a multi-faceted etiology. Although numerous studies on glaucoma using different animal models have been published, it is unwise to simply generalize the results of one model to all glaucomatous situations because of the differences in the anatomy and morphology of animal eyes in comparison with humans’. In this review, we highlight the differences in the trabecular meshwork (TM) tissue, lamina cribrosa (LC) region, optic nerve head (ONH) and the inner layer of the retina in mice, rats and monkeys. In comparison with humans, non-human primates show TM, retina and ONH that are anatomically almost identical. The rat model shows many similarities in the aqueous outflow pathway compared to humans. The mouse ONH lacks collagenous LC, and this finding is observed across different mouse strains. The tissue structure of the ONH in rodents is similar to that in humans, although the blood supply shows differences. The number of cells in the ganglion layer depends on the rodent strain. Despite some differences from humans, rodents are a good choice for studying different types of glaucoma, and the modeling method should be selected based on the experimental needs and the hypothesis being tested.

## 1. Introduction

Glaucoma is the second leading cause of blindness worldwide and has been estimated to affect nearly 66 million people [1], a number that is expected to increase to 80 million by 2020 [2]. It is a group of ocular disorders associated with optic nerve degeneration and retinal ganglion cell death, leading to visual field loss and eventual blindness [3]. Despite several efforts and studies, the pathophysiology of glaucoma is not clearly understood.

A variety of animal models have been used to mimic different glaucomatous situations, including mouse [4,5], rat [5,6], rabbit [7], pig [8], cat [9,10], dog [10,11] and monkey models [12,13]. However, each has its own disadvantages and drawbacks, especially with regard to differences in eye anatomy and morphology in comparison with humans [14]. These differences not only exist in the anterior eye segment, but also in the lamina cribrosa (LC) region, the optic nerve head (ONH) and in the inner layer of the retina. In this paper, we attempt to review the anatomical differences in the rodents and primates being used in glaucoma research.

## 2. Comparative Anatomy and Composition of the Trabecular Meshwork and Schlemm’s Canal

The trabecular meshwork (TM) is located at the iridocorneal angle overlying Schlemm’s canal (SC) and regulates the outflow of the aqueous humor [15]. Vision loss in most forms of glaucoma is often caused by elevated intraocular pressure (IOP), which occurs upon abnormally high resistance to aqueous humor drainage via the TM and SC [16].Studies have reported changes in the histological morphology and extracellular matrix components of the TM tissue in glaucomatous patients’ eyes [17,18,19].

Conventional light and electron microscopy studies have previously demonstrated that rats possess a similar aqueous humor outflow pathway with an ultrastructure resembling that of primates [20,21,22]. This includes the TM and a prominent single SC, connected to the episcleral vasculature by trans-scleral collector channels [23] (Figure S1B). Taking advantage of these anatomical similarities, a model of chronically-elevated IOP was built by injecting hypertonic saline into one of the episcleral radical veins [24]. The TM in the rat eye is, however, far less intensive than that in the human eye and extends up to the spaces of Fontana [21]. Although the rat TM may be less capable of generating a high flow resistance because it is only a few cell layers thick, we still could observe a statistical decrease in IOP after TM ablation treatment [25].

The mouse models, as well as rat models, share many similarities with human eyes in the morphology of the anterior chamber angle. The TM of the C57BL/6 mouse is a thin (only three to four trabecular lamellae), triangular-shaped wedge of tissue that encircles the anterior chamber, immediately adjacent to a well-developed and continuous SC [26,27] (Figure S1A). Some of the TM lamellae attach to the pectinate ligament (PL) spanning between the peripheral cornea and iris root [28]. In addition to the angle structures, the mouse developmental sequence for other structures is similar to that in humans [29]. Although the uveoscleral outflow, not the conventional outflow, is the main aqueous humor drainage pathway in young mice, accounting for approximately 80% of the total outflow, such difference in outflow pathway will diminish sharply with age. The aqueous humor dynamics in aged mouse is approaching that in early middle aged human [30]. The similarities above suggest that the mouse may be a good model to study aqueous humor outflow in primates and in glaucoma.

The anterior part of the monkey trabecular meshwork is nearly organized exactly as in the human eyes [31] (Figure S1C,D). Under electron microscopy, the layer next to the inner wall is observed to be considerably constant and continuous within the middle and posterior part, while the discontinuities correspond to those found in human eyes [32]. With respect to the topography of the endothelium [33,34], no differences have been found in the size, configuration and cellular components of the endothelial cells and in the intercellular relationships. The main difference in the appearance of the SC between human and monkey eyes is the large amount of septa in the latter [32]. In general, the TM tissue and the SC in monkey and human eyes seem to be almost identical. The human TM consists of a network of collagen beams covered by endothelium-like cells, with extracellular matrix occupying the spaces between the beams [35]. It has been reported that the mouse TM contains a net of elastic (EL) fibers that spans the scleral sulcus and tethers together the CM, choroid, TM and the inner wall of the SC [28]. This EL net is similar to that found in primates [36,37]. Different animal models also share some similarities in the compositions of the cytoskeletal proteins of the TMs. Immunohistological analysis revealed that vimentin is expressed in the TM tissue of the rat and human [38], and α-smooth muscle actin [28,38,39,40,41] is expressed in the TM tissue of the mouse, rat, monkey and human (Table 1).

## 3. Comparative Anatomy and Composition of the Lamina Cribrosa

Although numerous other species, including rabbits [42], pigs [43], cats [44] and dogs [45], develop an LC structure, the mouse ONH lacks similar connective tissue bundles at the level of the sclera [46,47,48] (Figure S2A). This finding is independent of the different mouse strains analyzed. While this differs from human eyes, studies in glaucomatous mice might help identify the role of the LC in optic nerve damage in glaucoma.

It should be noted that single collagen bundles are present and form an LC-like structure in place of a well-developed, collagenous LC in the rat ONH. Unlike the mouse models, the quantity of LC seems to vary across rat strains: the brown Norway rat [49] (Figure S2B) and the Long Evans rat [50] were reported to have a substantial LC, whereas only sparse LC bundles were found in the PVG hooded rat [51] and Wistar rat [52]. However, these differences from primates do not diminish the popularity and utility of rats for studying the pathophysiological mechanisms of axonal injury from elevated IOP and in glaucoma.

Monkeys, without a doubt, show well-developed collagen fibers, which make up a marked multi-layered LC and a flat disc [48,53,54,55] (Figure S2C). Light microscopy results reveal that the anterior portion of the monkey LC is an extensive structure that occupies almost the entire ONH [56]. Fenestrated connective tissue sheets derived from the sclera form the main support for the posterior portion of the LC. The sheets consist of a dense collagenous tissue with pigment cells, while they often show elastic fibers in humans. The different values for the LC diameter are shown in Table 2.

Numerous papers have demonstrated the macromolecular components of the extracellular matrix of the LC. The cribriform plates are made up of a core of elastin fibers with collagen types I, III, VI and laminin and collagen type IV (basal membranes) along the laminar beam margins, astrocytes and vessels. This composition has so far only been studied in rats [49,52,58], monkeys [53,59,60,61] and humans [62,63,64,65]. Besides the above macromolecular components, chondroitin and dermatansulfate proteoglycans were observed in immunofluorescent and immunohistochemical analyses in the rodent [49], monkey [66] and human LC [67,68,69].

We can see that rodents and primates show similarities in the composition of the LC. However, more accurate quantitative techniques have shown that the amount of collagen type VI varies across species, with weakly-stained collagen type V and VI in the normal human LC [70] and intensely-stained collagen type VI in the normal rat LC [49,52].

## 4. Comparative Anatomy of the Central Retinal Vessels

In rodents, the central retinal artery (CRA) primarily arises from the ophthalmic artery, branches into two posterior ciliary arteries and obliquely enters the optic nerve (ON) through the scleral ring and choroid towards the center of the ONH [47,71,72] (Figure S3A,B). A peculiar and frequent finding is the presence of intra-arterial cushions [73,74] in rodent arteries before ramification, including the ophthalmic artery. In the mouse ophthalmic artery, a v-shaped cushion [47] is regularly located before the branching of the CRA that might functionally regulate the blood flow in this specific region [75]. Nevertheless, such structures have not been found in the human ophthalmic artery.

The central retinal vein (CRV) of the rat eye originates from the confluence of the major retinal veins at the ONH and receives blood from several capillary beds (Figure S3B). The CRV travels closer [47] and more posterior [72] to the ON than the artery and communicates with the choroidal venous system [72,76].

In primates, the CRA also branches off the ophthalmic artery, but enters the ON posteriorly to the LC in comparison to that in rodents [77] (Figure S3C). The CRV lies adjacent and parallel to the CRA with a distinct centripetal pattern through the LC. The CRV is connected with the choroidal circulation in the prelaminar region [78]. Both vessels branch in the center of the ONH, forming the main retinal vessels.

## 5. Comparative Anatomy of the Optic Nerve Head Blood Supply

Because vascular mechanisms may also contribute to glaucomatous optic neuropathy, it is important to determine optic nerve head perfusion in different glaucomatous models [78,79]. On the basis of the results obtained with corrosion cast preparations and serial sections, we summarize the blood supply of the ONH in different species (Table 3).

In the mouse, both corrosion cast preparations and serial sections reveal that the non-myelinated portion of the ON is supplied exclusively by the CRA without forming any branches [47] (Figure S3A). Neither the choroid nor the pial vessels contribute to this region. Other than the arterial Zinn-Haller ring inhuman eyes, there are no additional arterial circles in mouse eyes.

The microvasculature of the rat ONH bears several similarities to that of the mouse, with most of the vessels derived from the retina, but the posterior ciliary arteries and some branches emanating from the choroid vessels are also found within the ONH [71,72,76] (Figure S3B). In contrast to mouse eyes, the CRA in rat eyes supplies blood not only tithe surface nerve fiber layer, but also tithe prelaminar, laminar and retrolaminar regions [76]. The venous system of the rat ONH includes CRV and optic sheath veins. The association of nerve sheath veins with the choroid represents an important difference from primates [72]. There are controversial opinions regarding the presence of the arterial Zinn-Haller ring in rat eyes: some authors have observed a similar arterial circular structure [72], while others have reported that they could not find it [76]. The disagreement might be caused by the difficulty in visualization of the ONH angioarchitecture; therefore, additional comparative studies are needed to investigate ONH blood supply in rats.

Hayreh conducted a series of studies [78,80,81,82] to update the knowledge of the vascular supply of the ONH in primates (Figure S3C). In his recent report [81], he describes the arterial supply of the ONH based on four regions from the anterior to the posterior aspect: (1) the surface nerve fiber layer is supplied by retinal arterioles; in some cases, the temporal region may instead be supplied by the posterior ciliary artery (PCA) circulation; (2) the prelaminar region is supplied by the fine centripetal branches from the peripapillary choroid; (3) the LC region is entirely supplied by centripetal branches from the short PCAs or the Zinn-Haller circle; (4) the blood supply to the retrolaminar region is provided by both the pial vascular plexus and the branches of CRA. The venous drainage of the ONH is relatively simple; it essentially occurs via the CRV, except that the prelaminar region also drains into the peripapillarychoroidal veins.

## 6. Comparative Anatomy of the Ganglion Cell Layer

Despite the basically consistent gross morphology of the retinal layers in all animals used for glaucoma research, some differences can be observed in the inner retina, mainly in the appearance of the ganglion cell layer (GCL). There are three important types of cells in the retina: (1) the photoreceptor cells (rods and cones), which detect and convey information to secondary neurons; (2) the ganglion cells, whose dendrites extend into the inner plexiform layer, and axons to make up the optic nerve; and (3) the displaced amacrine cells, whose specific role in the GCL has not yet been determined. We focus our attention mainly on the latter two cell types.

The mouse retina is strongly rod-dominated, with a higher density than that in monkeys [83,84]. At the level of the GCL, the numbers of ganglion cells are highly variable among different mouse strains, ranging from 32,000 to 87,000 [85,86]. Genetic factors play a vital role in controlling this variation [86]. One study of the C57/BL6 mouse retina shows that the fraction of ganglion cells and displaced amacrine cells is 41% ± 4% and 59% ± 4% in the ganglion cell layer [83].

The strain variations in the numbers of ganglion cells are also remarkable in rats, with pronounced differences in the number of optic nerve fibers between pigmented rats (around 70,000) and albino rats (more than 100,000) [87,88]. Based on the results of different studies, the total number of ganglion cells in various rat strains is about 70,000 to 120,000 [87,88,89,90,91]. The amacrine cells account for approximately 50% of the cells in the ganglion cell layer [92], and the density changes with eccentricity, ranging from 1700 centrally to 600 cells per mm^2^ in the periphery [93].

The number of ganglion cells in non-human primate eyes (cynomolgus monkey: 850,000 to 1,300,000 [94]; *Cercopithecus aethiops*: 1,220,000 [95]; rhesus monkey: 850,000 to 1,500,000 [94,96]; cebus monkey: 1,340,000 to 1,400,000 [97]) is comparable to that in human eyes (700,000 to 1,500,000 [98]). The distribution of displaced amacrine cells in the monkey GCL is distinctly non-uniform in different regions [99]: the concentration is lower (5% of all neurons) in the foveal region and higher in the nasal (up to 30%) and temporal regions (50%). The human retina shows a similar distribution pattern, with 3% displaced amacrine cells in the foveal region and up to 80% in the peripheral part [98].The quantitative data of the ganglion cell layer in different species is summarized in Table 4.

## 7. Summary of the Rodent and Primate Models Commonly Used for Glaucoma Research

Since there are several types of glaucoma, we have summarized the mode and mechanisms employed indifferent animal models in relation to the human clinical glaucoma classification (Table 5).

## 8. Conclusions

A comprehensive evaluation of rodent and primate models depends on the degree of similarity between the model and the human condition, as well as practical and economic considerations.

Non-human primates are the most appropriate animals for studying glaucoma because of the close phylogeny and high homology with humans. They have an almost anatomically identical TM, retina and ONH. However, we cannot ignore the disadvantages posed by the strict ethical considerations, the high cost of obtaining these animals, the need for special housing facilities and the limited supply. These limitations restrict their utility in large-scale experiments.

As mentioned above, the mouse ONH lacks a collagenous LC that may either provide significant protection for optic nerve fibers or actively contribute to axonal damage. Therefore, mouse models cannot be used for LC-specific functional investigations. However, some researchers have found that experimental IOP elevation can lead to optic nerve damage and RGC loss in mice, which suggests that the LC is not an essential influencing factor for the process of glaucoma [119,120,121]. The composition of mouse and human ONH is similar, although the two show clear differences in blood supply. In addition, the number of cells in the ganglion layer differs across mouse strains. Since mice are small animals, the size of their eyes makes it difficult to simulate the complex surgeries performed in humans. Despite these disadvantages, the mouse is one of the most widely-used models for glaucoma. The reasons include the high degree of conservation between mice and human genomes, the availability of mature technology for genetic manipulation [122] and the convenience of breeding the animals. Mice are inexpensive, and their eyes are easy to obtain, so the experimental sample numbers can be large.

In contrast to other non-primate models, the rat shares similar anatomical and developmental characteristics of the anterior chamber with humans, especially in the aqueous outflow pathway. Similar to mice, strain differences seem to play an important role when investigating the role of the LC. The vascular supply of the ONH should be taken into account when comparing findings with the human situation. The advantages of using rats also include easy laboratory support, availability of genetic manipulation tools and ease of using large samples.

Since the mechanisms underlying glaucoma differ among animal models [123], the results obtained from a particular model should not be simply generalized and should be interpreted within the context of that model. Rodents are a good choice for studying different types of glaucoma, and modeling methods should be selected on the basis of the experimental needs and the hypothesis being tested.

## Figures and Tables

**Table 1:** Composition of the trabecular meshwork (TM) in different species.

Species	Composition of TM		
	Elastic Fibers	Vimentin	α-Smooth Muscle Actin
Mouse	+	N/A	+
Rat	N/A	+	+
Monkey	+	N/A	+
Human	+	+	+

+ = positive expressed; N/A = not reported.

**Table 2:** Presence and diameter of the lamina cribrosa (LC) in different species.

Species	Presence of LC	Diameter of LC/Optic Nerve at the Level of the Sclera
Mouse	–	193 ± 8 μm
Rat	(+)	201 ± 1 μm
Monkey	+	1717 ± 21 μm
Human [57]	+	1580 ± 210 μm

– = not present; (+) = faintly present; + = well developed.

**Table 3:** Blood supply to the optic nerve head region.

Species	Central Retinal Artery	Choroid	Pial Vessels
Mouse	+	−	−
Rat	+	(+)	(+)
Monkey	+	+	+
Human	+	(+)	+

− = no, (+) = possible, + = clear.

**Table 4:** The quantitative data of the ganglion cell layer. GCL, ganglion cell layer.

Species	Ganglion Cell Number	Percentage of Amacrine Cells of the GCL
Mouse	32,000 to 87,000	59%
Rat	70,000 to 120,000	50%
Monkey	850,000 to 1,500,000	Fovea 5%, nasal 30%, temporal 50%
Human	700,000 to 1,500,000	Fovea 3%, peripheral 80%

**Table 5:** Summary of rodent and primate models commonly used for glaucoma research.

Glaucoma Type	Species	Method	Reference
POAG	Mouse	Transgenic mouse with myocilin mutation	[100]
		Transgenic mouse with type I collagen mutation	[101,102]
	Rat	Application of topical dexamethasone to rat eyes	[103]
	Monkey	Laser photocoagulation of entire TM	[104]
		Intracameral injection of sterilelatex microspheres	[105]
		Intracameral injection of autologous fixed red blood cells	[106]
PACG	Mouse	Transgenic mouse with Vav2/Vav3 deficiency	[107]
		Laser photocoagulation of the episcleral and limbal veins	[108]
		Cauterization of episcleral veins	[109]
	Rat	Cauterization of episcleral veins	[110]
		Ligation of episcleral veins	[111]
		Laser photocoagulation of TM and episcleral veins	[112]
		Episcleral veins injection of hypertonic saline	[24]
		Intracameral injection of hyaluronic acid	[113]
PCG	Mouse	Transgenic mouse with CYP1B1 mutation	[114]
		Transgenic mouse with CYP1B1 and Tyr mutation	[115]
	Rat	Spontaneous inheritance of WAG strain	[116]
		Spontaneous inheritance of RCS-rdy-strain	[117]
Pigmentary	Mouse	DBA/2J strain	[118]

POAG: primaryopenangle glaucoma; PACG: primary angle closure glaucoma; PCG: primary congenital glaucoma.

## Supplementary Materials

The supplementary materials can be found at www.mdpi.com/2411-5150/1/1/4/s1.
