# Supplementary material for: Comparative Anatomy of the Trabecular Meshwork, the Optic Nerve Head and the Inner Retina in Rodent and Primate Models Used for Glaucoma Research
Source: Vision (Basel). 2016 Jul 18;1(1):4. doi: 10.3390/vision1010004 (PMC6848998; doi:10.3390/vision1010004)
Supplement: Supplementary file 1 [file vision-01-00004-s001.pdf]

Supplementary Materials: Comparative Anatomy of the Trabecular Meshwork, the Optic Nerve Head and the Inner Retina in Rodent and Primate Models Used for Glaucoma Research

Liwen Chen, Yin Zhao and Hong Zhang

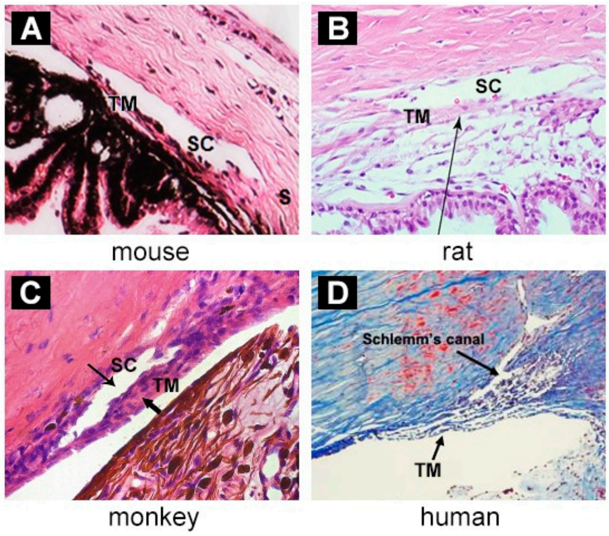

Figure S1. The trabecular meshwork and Schlemm's canal of different animal models. (**A**) The TM of the C57BL/6 mouse is a thin (only three to four trabecular lamellae), triangular-shaped wedge of tissue, immediately adjacent to a well-developed and continuous SC [124]; (**B**) The TM of the Wistar rat also possesses a few cell layers thick TM and a prominent single SC [125]; The TM tissue and the SC in monkey (**C**) and human (**D**) eyes seem to be almost identical under light microscopy [31,126]. TM: trabecular meshwork; SC: Schlemm's canal. All images have been reprinted with permission from Association for Research in Vision and Ophthalmology, Academic Press and Pergamon Press.

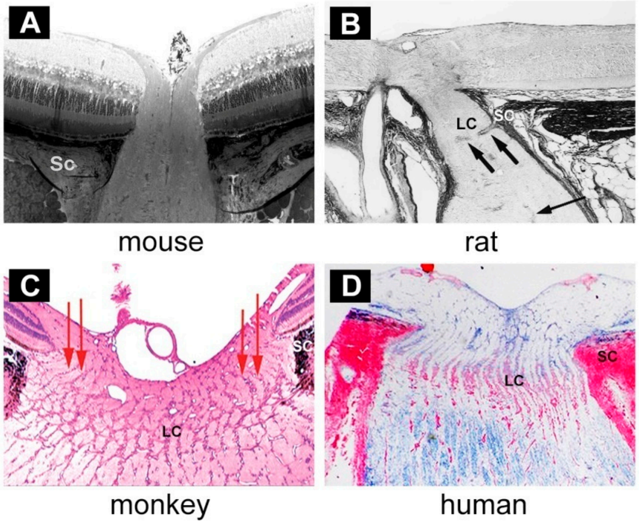

Figure S2. The optic nerve head and lamina cribrosa of different animal models. (**A**) The mouse ONH lacks LC structure at the level of the sclera [47]; (**B**) The Brown Norway rat ONH has a substantial LC structure at the level of the sclera [127]; Monkeys (**C**) and human (**D**) ONH both show well-developed collagen fibers at the level of the sclera, which make up a marked multi-layered LC structure [128,129]; Sc: sclera; LC: lamina cribrosa. All images have been reprinted with permission from Association for Research in Vision and Ophthalmology and Churchill Livingstone.

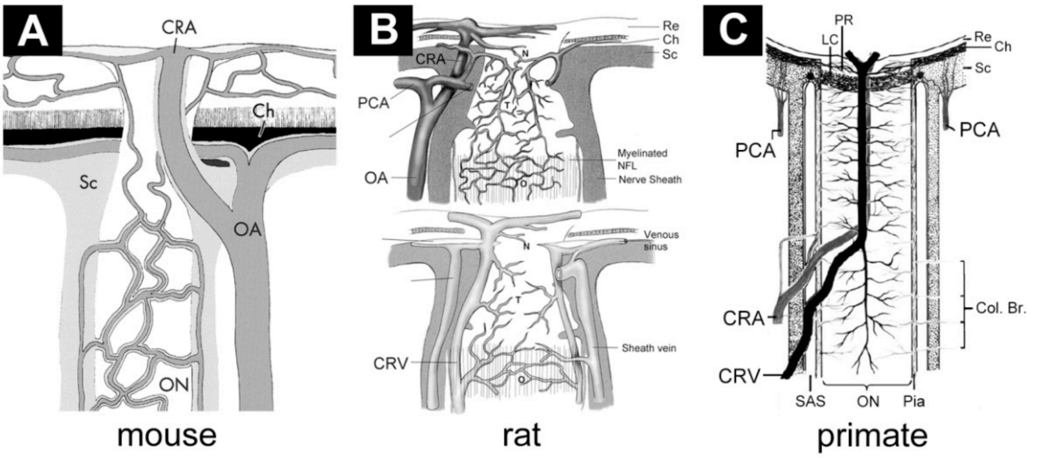

Figure S3. The schematic drawings of the optic nerve head blood supply of different animal models. In mouse (**A**) and rat (**B**), the CRA primarily arises from the OA, branches into two PCA, and obliquely enters the ON through the scleral ring and choroid towards the center of the ONH [47,72]; In primate (**C**), the CRA branches off the OA, but enters the ON posteriorly to the LC in comparison to that in rodents. The CRV is connected with the choroidal circulation in the prelaminar region [77]. CRA: central retinal artery; CRV: central retinal vein; PCA: posterior ciliary artery; OA: ophthalmic artery; Sc: sclera; Ch: choroid; Re: Retina; LC: lamina cribrosa; PR: prelaminar region. All images have been reprinted with permission from Association for Research in Vision and Ophthalmology and Pergamon Press.

© 2016 by the authors; licensee MDPI, Basel, Switzerland. This article is an open access article distributed under the terms and conditions of the Creative Commons Attribution (CC-BY) license (http://creativecommons.org/licenses/by/4.0/).
